# Biomechanical effects of morphological variations of the cortical wall at the bone-cement interface

**DOI:** 10.1186/s13018-016-0405-y

**Published:** 2016-07-01

**Authors:** Chun-Lin Zhang, Guo-Qi Shen, Kun-Peng Zhu, Dong-xu Liu

**Affiliations:** Department of Orthopaedic Surgery, the Tenth People’s Hospital Affiliated to Tongji University, #301 Yan-chang Middle Road, Shanghai, 200072 China; Department of Orthopaedic Surgery, Changshu Second People’s Hospital, Changshu, 215500 China; Department of Orthopaedic Surgery, The Sixth People’s Hospital Affiliated to Shanghai Jiaotong University, Shanghai, 200233 China; Orthotek Lab, School of Mechatronics Engineering and Automation, Shanghai University, No. 149, Yanchang Rd, 200072 Shanghai, People’s Republic of China

**Keywords:** Interface, Bone cement, Morphology, Biomechanics

## Abstract

**Background:**

The integrity of bone-cement interface is very important for the stabilization and long-term sustain of cemented prosthesis. Variations in the bone-cement interface morphology may affect the mechanical response of the shape-closed interlock.

**Methods:**

Self-developed new reamer was used to process fresh pig reamed femoral canal, creating cortical grooves in the canal wall of experimental group. The biomechanical effects of varying the morphology with grooves of the bone-cement interface were investigated using finite element analysis (FEA) and validated using companion experimental data. Micro-CT scans were used to document interlock morphology.

**Results:**

The contact area of the bone-cement interface was greater (*P* < 0.05) for the experimental group (5470 ± 265 mm^2^) when compared to the specimens of control group (5289 ± 299 mm^2^). The mechanical responses to tensile loading and anti-torsion showed that the specimens with grooves were stronger (*P* < 0.05) at the bone-cement interface than the specimens without grooves. There were positively significant correlation between the contact area and the tensile force (*r*^2^ = 0.85) and the maximal torsion (*r*^2^ = 0.77) at the bone-cement interface. The volume of cement of the experimental group (7688 ± 278 mm^3^) was greater (*P* < 0.05) than of the control group (5764 ± 186 mm^3^). There were positively significant correlations between the volume of cement and the tensile force (*r*^2^ = 0.90) and the maximal torsion (*r*^2^ = 0.97) at the bone-cement interface. The FEA results compared favorably to the tensile and torsion relationships determined experimentally. More cracks occurred in the cement than in the bone.

**Conclusions:**

Converting the standard reaming process from a smooth bore cortical tube to the one with grooves permits the cement to interlock with the reamed bony wall. This would increase the strength of the bone-cement interface.

## Background

Cemented prostheses are widely used because they generally remain firmly fixed during the early postoperative stage [1–5]. However, aseptic loosening is an important long-term complication of prosthesis and is in part responsible for the increasing number of patients requiring revision surgery. Clinical evidence suggests that failures and the incidence of loosening along the bone-cement interface are prevalent [6, 7]. Initial fixation strength of the prosthetic components is the main factor that influences the long-term service life of the implant. The integrity of bone-cement interface is very important for the stabilization and long-term sustain of artificial prosthesis [4].

Loosening is considered a complicated function of both mechanical and biological factors [8]. Although the incidence and progression of loosening in cemented joint replacements is poorly understood, it is generally accepted that trabecular bone resorption at the bone-cement interface contribute to failure of the bone-cement interface [9]. The fixation strength of cemented artificial joint is dependent on good interlocking of the bone-cement interface. Bone-cement interface interlocking strength is positively correlated with contact area and interlocking depth of interface. By increasing the bone-cement interface contact area and interlocking depth, artificial joint replacement surgery can be ensured to get better initial fixation strength, resulting in extended life of artificial joint and reduced complications [10, 11]. Variations in the bone-cement interface morphology may affect the mechanical response of the shape-closed interlock. Few studies to the morphology of bone-cement interface have been reported [12–15]. In the present study, we postulated that using self-developed new reamer to create some superficial glossy grooves in the medullary canal wall permits increasing the interlock area of the bone-cement interface. This morphology change of the bone-cement interface may improve the mechanical strength and potential longevity to the bone-cement-implant component. The primary objective of this study was to quantify the contact area and strength of the bone-cement interface and to analyze morphological changes of the components associated with the morphological variations of the cortical wall.

## Methods

This study has been granted an exemption from requiring ethics approval by the ethic committee of the Tenth People’s Hospital Affiliated to Tongji University.

### Specimen and model preparation

**Fig. 1:**
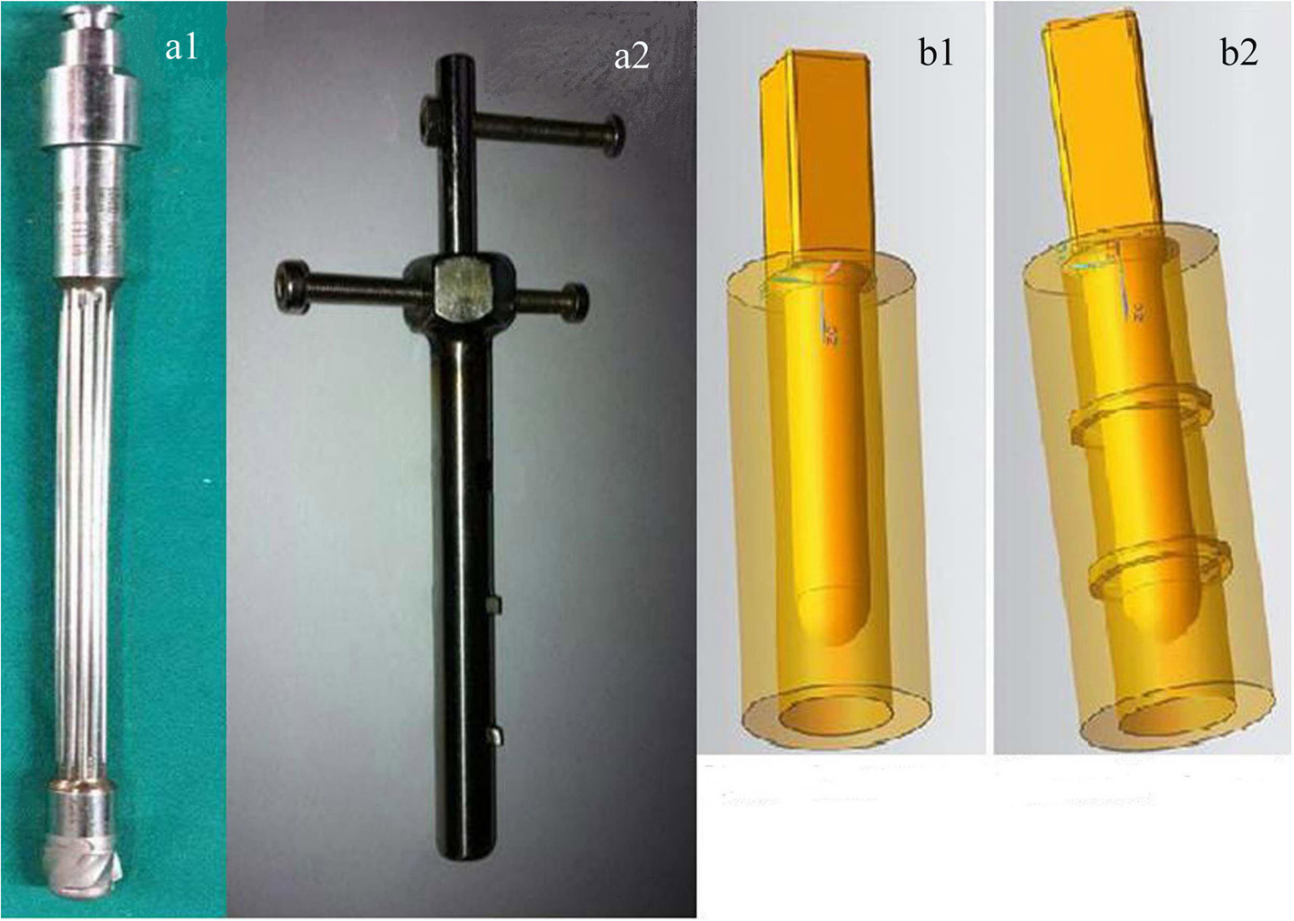
The reamers and the relevant diagram of 3-D components (**a1** traditional reamer, **a2** self-developed reamer, **b1** diagram of component without grooves, **b2** diagram of component with grooves)

After removing the surrounding soft tissues, the muscles and the femoral condyles of the fresh pig’s femurs which were purchased from the supermarket, 40 samples were cut. The length of the model femur bone was 60 mm each. The 64-slice spiral computed tomography (CT) was used to scan the femur samples. Forty samples were randomly assigned to the experimental group and the control group, with each group 20 samples. The bone marrow and most of cancellous bone of all samples were removed. In control group, the traditional reamer (Link Company, Germany) was used to ream the samples. In experimental group, the traditional reamer was used to ream the samples firstly, then the self-developed reamer which had two cutting teeth (Fig. 1) was used to create two cortical grooves in the canal wall. The two grooves respectively located at 20 and 40 mm of the proximal femoral shaft, and both were 2 mm wide and 2 mm deep. This treatment of the inner wall of the medullary cavity was not performed in the control group which was reamed only by the traditional reamer. After reaming, pulsed lavage was used to clean bone particles inside the medullary canal.

**Fig. 2:**
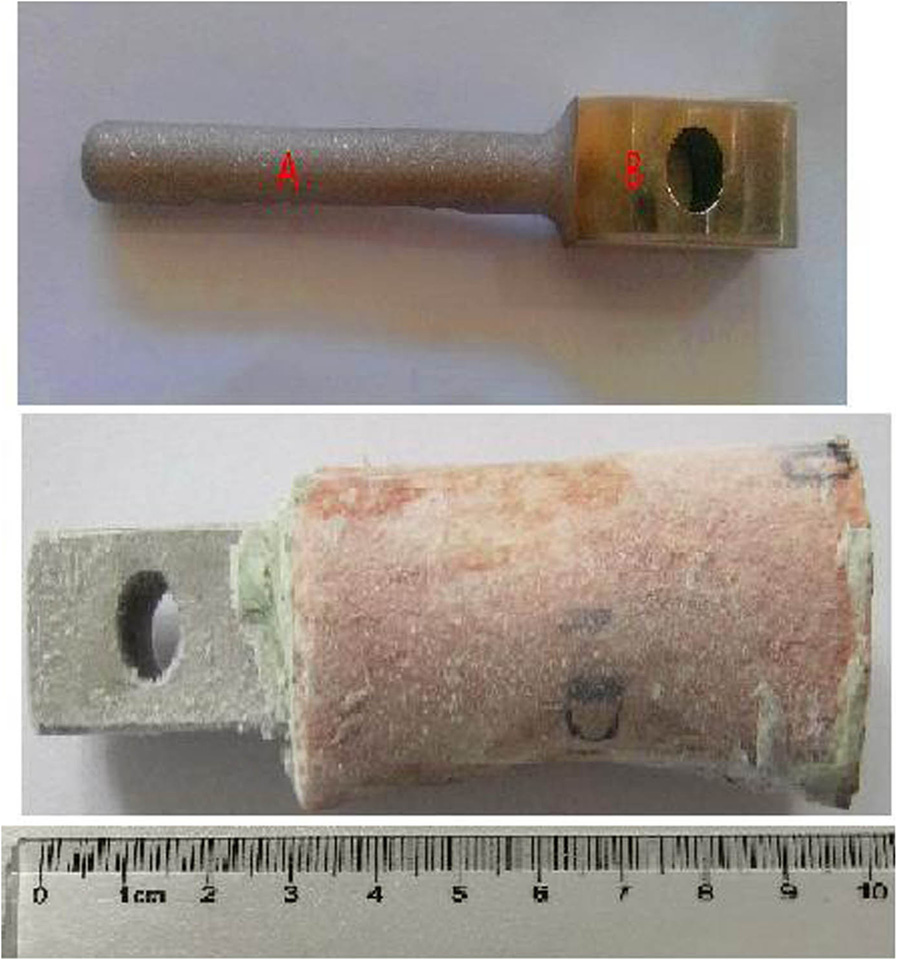
The model of bone-cement-prosthesis component

The CoCrMo prosthesis was inserted to the femur after cement importing (Fig. 2). The stem of CoCrMo prosthesis was cylindrical with 12 mm diameter, 50 mm length, and with a distal smooth cambered surface. A 200-N constant pressure was applied to the fixed rod of the prosthesis until the cement fully solidified in 10 min. The thickness of cement was 3 mm due to the diameter of the femoral canal and the properties of the prosthesis. All operations were done at temperature of 21 ± 1 °C and humidity of 55 ± 5 %.

### Detection of micro-computed tomography

All specimens were scanned with a micro-computed tomography (micro-CT) scanner (phoenix-v|tome|x m, General Electric Company, USA) to document cement and bone morphology before biomechanical testing. Models were vertical inverted to place on fixed jaw of instrument. Each scanning was at clockwise rotation with 0.19° and voltage of 240 kV, current of 200 μA, and resolution of 45 μm. The average scanned time was 90 ± 5 min. We used the DATOSX software (General Electric Company, USA) after scanning for image acquisition and reconstructed visualization. Three-dimensional morphology measurements were made using the VGstudio software (Volume Graphics, Germany), which was based on a single threshold of CT gray value of the bone, cement, and artificial prosthesis, including the total volume (TV), the volume of cement (CV), the bone volume (BV), the pore volume (PV), and the contact area of the bone-cement interface (CA). The porosity was the ratio of the pore volume to the total volume of the specimen (PV/TV). The bone volume fraction was the ratio of the bone volume to the total volume (BV/TV). The metal prosthesis may have great image effects on cement and bone during the image reconstruction due to the prosthesis was a cobalt-chromium-molybdenum material. Therefore, we must extract the images of the metal prosthesis from models, and then the bone and cement for analysis and calculation, to avoid the effects caused by metal prosthesis.

### Biomechanical testing

After micro-CT detection, all 40 specimens were randomly divided into experimental group and control group, including 10 specimens of tensile experimental group, 10 specimens of tensile control group, 10 specimens of torsion experimental group, and 10 specimens of torsion control group. The biomechanical tests of all specimens were performed by the USA pull torsional biomechanical testing machine (dynamic displacement ±50 mm; dynamic axial load ±14.2 kN; static torsion angle ±40°; static torque 200 Nm).

The test specimens were fixed vertically on the biomechanical testing machine. Before testing, the samples were adapted under the loading from −100 to 100 N and the frequency of 2 Hz. The initial load of the tensile testing was performed vertically upward to 100 N and 2 mm/min of displacement to implant fixation rod. In the torsion group, a similarly initial load that perpendicular to the longitudinal axis of the model was performed, 5°/min of rotational rate. When the specimens showed cortical bone fractures, separation of the bone-cement interface or cement-prosthesis interface, and fracture of prosthesis, which were considered model failure. The maximal load was the loading of model failure.

### Finite element modeling of the bone-cement-prosthesis composite

FEA models were created using micro-CT scans of specimens. The FEA meshes of the two models with and without grooves were created by meshing using ANSYS14.0 program. The initial material properties of the models were considered to be linear elastic and isotropic. Young’s modulus [16] and Poisson’s ratio of the femur, cement, and prosthesis were set to 16.7, 3.0, 200 Gpa, and 0.3, respectively. The bone properties were based upon micro-CT gray scale values, which were converted to equivalent HA-densities using a calibration phantom. The assumption of a linear relationship between the HA-density and the Young’s modulus resulted in Young’s moduli ranging from 0.1 to 20,000 MPa for the bone (*n* = 0.3). In the tension test, the load was added to the top of the prosthesis pointing downward vertically to simulate the biomechanical experimental load by an axial strength of 2.4 kN. Similarly, in the torsion test, a torque of 40 Nm was added to the top of the prosthesis vertically.

### Statistics

The data were analyzed descriptively using the arithmetic mean, standard deviation (SD), minimum value, and maximum value. The skewness and kurtosis coefficient were used to analyze the distribution of data. Paired *t* tests were used to determine if the data was normally distributed, otherwise, the rank test was used. *P* < 0.05 was considered statistically significant. Linear regression analysis was used to determine correlations between average contact area and tensile strength, maximal torsion, and porosity. The results of biomechanical experimental tests and FEA were compared. All data analyses were performed using SPSS 18.0 (SPSS, Chicago, Illinois).

## Results

### The morphology of bone-cement interface (micro-CT)

**Fig. 3:**
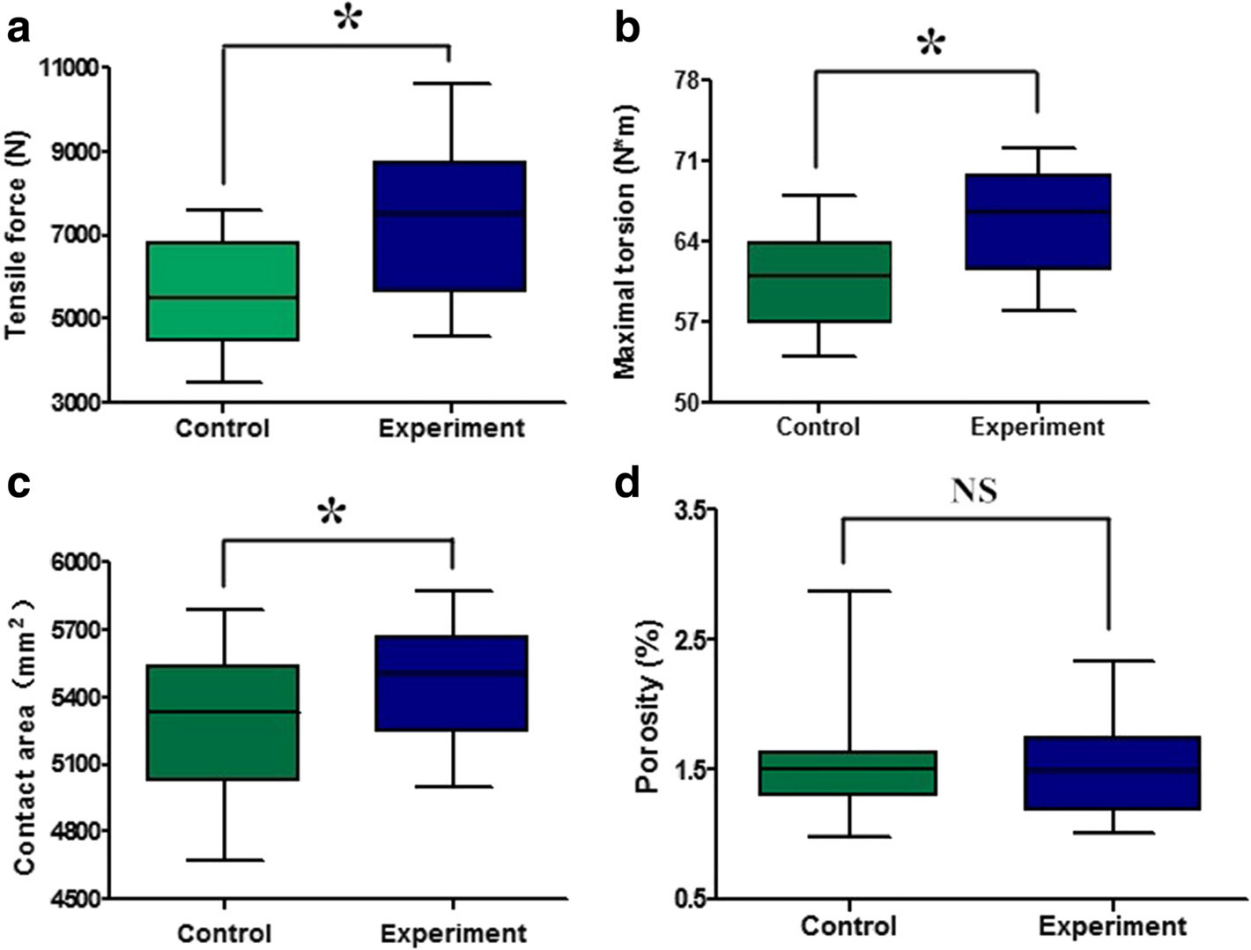
Tensile strength of the experimental group was significantly higher than the control group (**a**). Maximum torque in the experimental group was significantly higher than the control group (**b**). The contact area of bone-cement interface in the experimental group was significantly higher than the control group (**c**). There was no significantly different between the porosity of experimental and control groups (**d**)

Compared with the control group, bone cement in the specimens of experimental group had better penetration and better interlock between cement and bone, due to annular grooves of the inner wall of the bone medullary canal. The contact area of the bone-cement interface was greater (*P* < 0.05) for the experimental group (5470 ± 265 mm^2^) when compared to the specimens of control group (5289 ± 299 mm^2^). The volume of cement for the experimental group (7688 ± 278 mm^3^) was greater (*P* < 0.05) when compared to the specimens of control group (5764 ± 186 mm^3^). However, the porosity for the experimental group (1.50 ± 0.382 %) was similar (*P* > 0.05) to the control group (1.59 ± 0.496 %). There was no significant difference in the bone volume fraction (BV/TV) (*P* > 0.05) between the experimental group (0.493 ± 0.019) and control group (0.495 ± 0.031) (Fig. 3).

### Mechanical properties

**Fig. 4:**
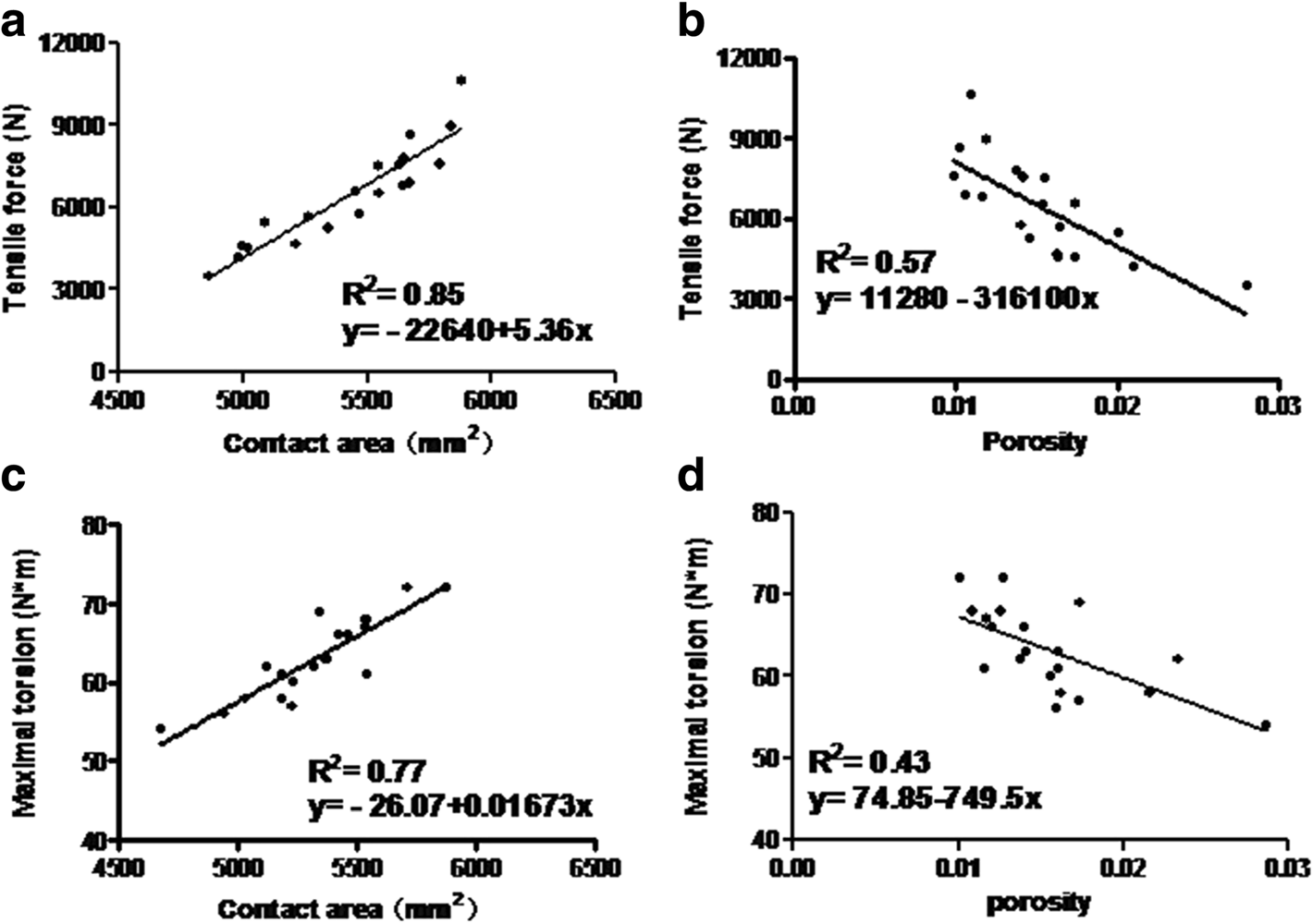
Linear regression models: tensile stress and contact area, *r* ^2^ = 0.85 (**a**); tensile stress and porosity, *r* ^2^ = 0.57 (**b**); maximum torque and contact area, *r* ^2^ = 0.77 (**c**); maximum torque and porosity, *r* ^2^ = 0.43 (**d**); *P* < 0.05

**Fig. 5:**
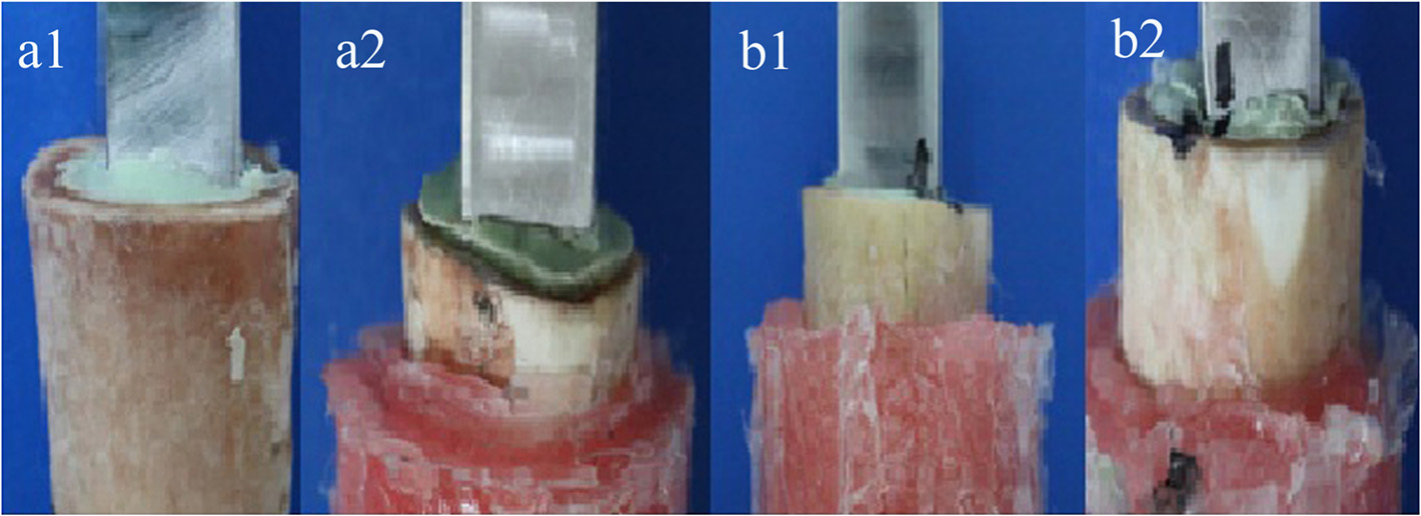
The responses after biomechanical test: before tensile test (**a1**), after tensile test—the stretched displacement occurred at the bone-cement interface of all the samples and the cement-prosthesis interface did not have a displacement (**a2**); before torsion test (**b1**), after torsion test—cortical bone fractures occurred in the samples, and significant loosening of the bone-cement interface was observed, while loosening of the cement-prosthesis interface was not observed (**b2**)

Under the loading of 2400 N, there was no significant difference of the average peak stress and the total deformation, respectively, for the components with grooves and the components without grooves. It showed that the creation of grooves in the intramedullary canal did not affect the integrity of the femur and jeopardize the strength of the femur either. The mechanical responses to tensile loading showed that the specimens of experimental group (7337 ± 1825 N) had stronger force (*P* < 0.05) at the bone-cement interface than the control group (5564 ± 1359 N). The anti-torsion capability was greater (*P* < 0.05) for the experimental group (65.70 ± 4.83 Nm) when compared to the control group (60.60 ± 4.43 Nm) (Fig. 3). There were positively significant correlations between the contact area and the tensile force (*r*^2^ = 0.85) and the maximal torsion (*r*^2^ = 0.77) at the bone-cement interface. Furthermore, the cement volume was strongly and significantly correlated with tensile force and maximal torsion, respectively (*r*^2^ = 0.90, *P* < 0.05 and *r*^2^ = 0.97, *P* < 0.05, Fig. 4). The relationships between the porosity and the tensile force and the maximal torsion were observed (*r*^2^ = 0.57, *P* < 0.05 and *r*^2^ = 0.43, *P* < 0.05, Fig. 4). In the tensile testing, the failure of all specimens (20/20) including the experimental group and the control group occurred at the bone-cement interface. There was a macroscopic displacement between the bone and cement. In the rotary testing, the failure of 17 specimens (17/20) occurred at the bone-cement interface (Fig. 5). More cracks occurred in the cement than in the bone.

**Fig. 6:**
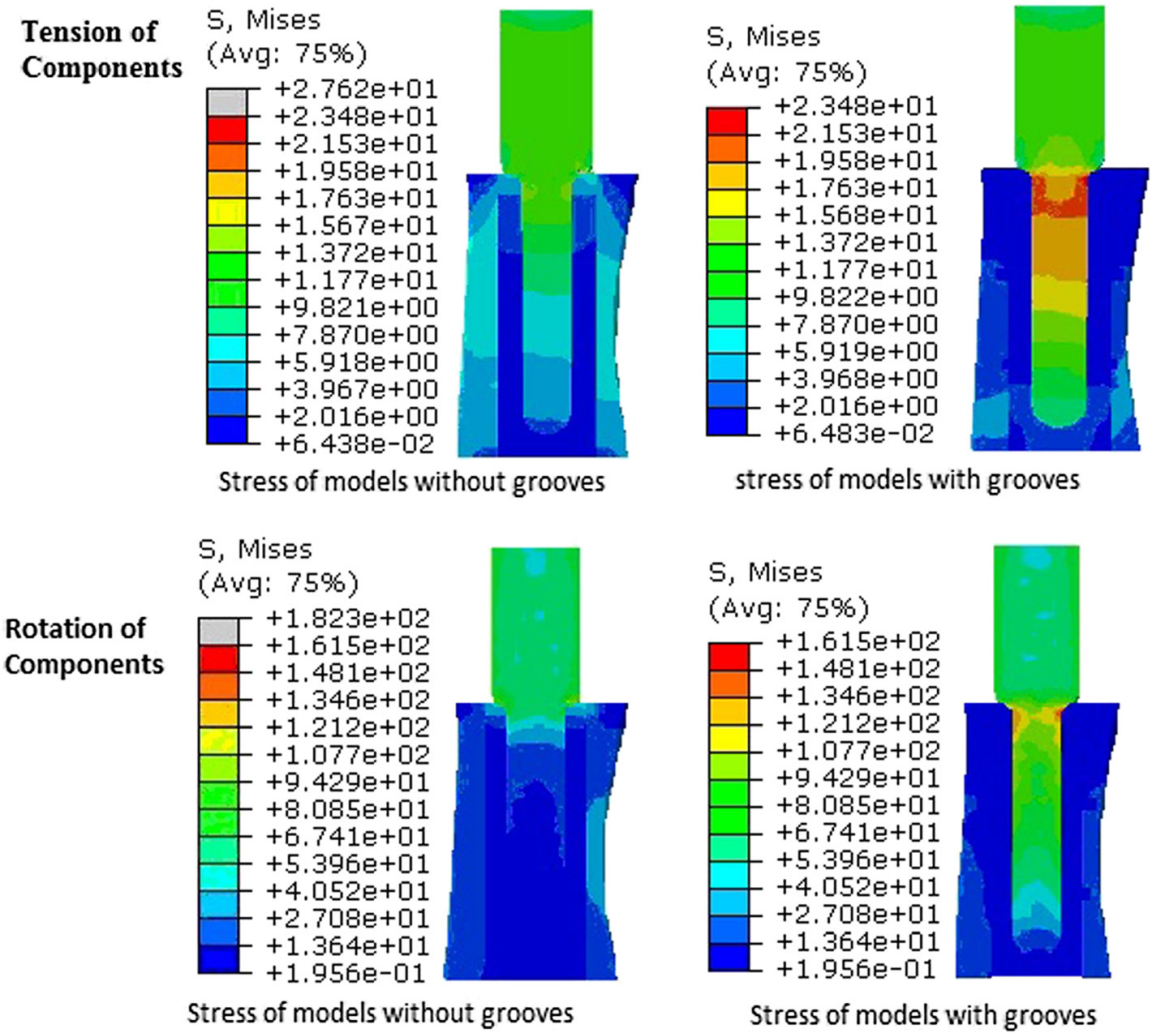
Under the tension and rotation loads, the von Mises stress in the components of the experimental group was lower than that in the components of the control group

**Fig. 7:**
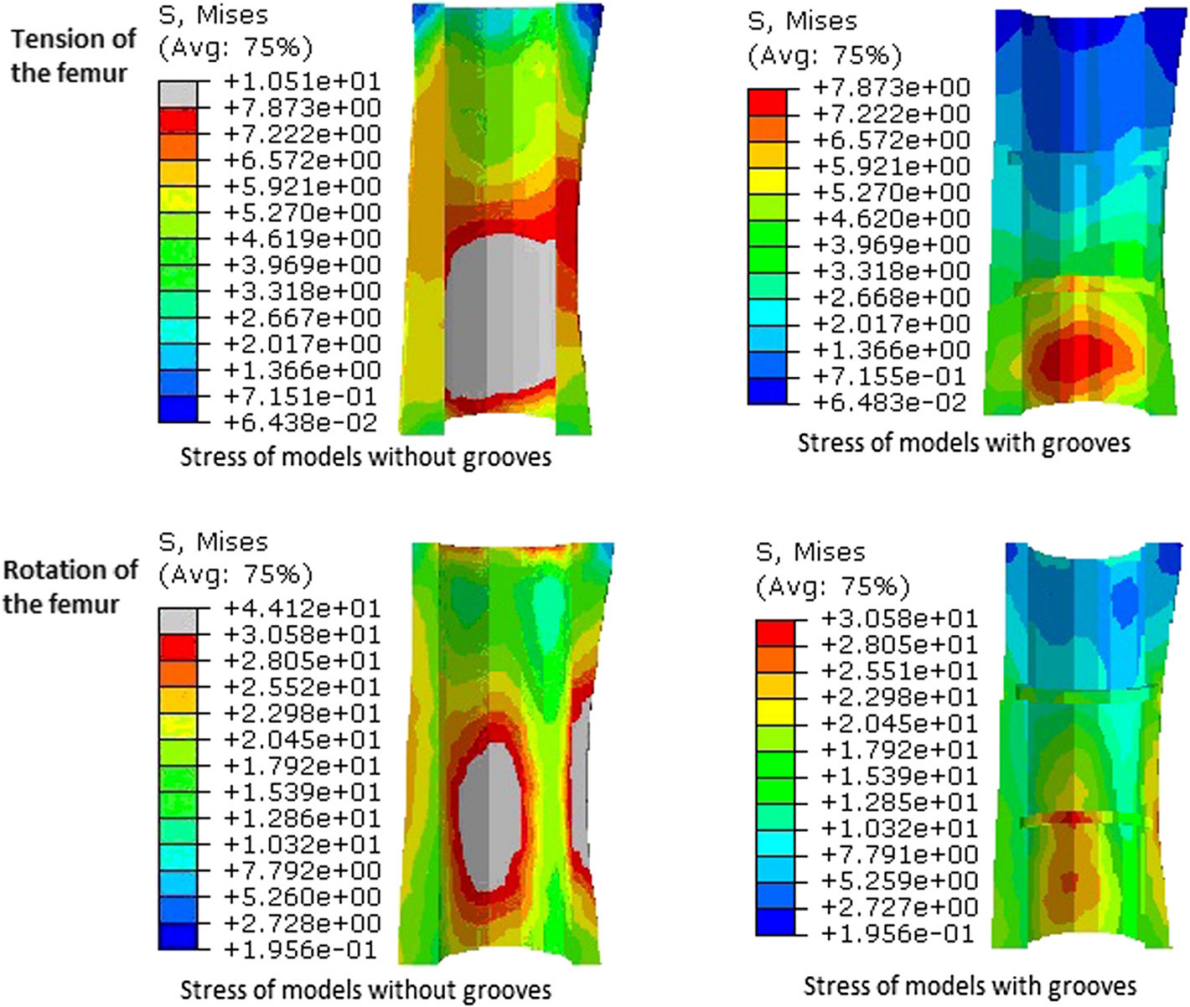
Under the tension and rotation loads, the von Mises stress in the femur of the experimental group was lower than that in the femur of the control group

In FEA, under the tension and torsion loads, the peak stresses of the components with grooves (23.48 and 161.5 MPa, respectively) were both lower than those of the components without grooves (27.62 and 182.3 MPa, respectively) (Fig. 6). The peak stresses of the femur of the experimental group were also lower than that of the control group under both tension and torsion loads (Fig. 7). Although stress concentration inside the grooves was observed, those peak stress values (around 7.9 MPa under tension load and 30 MPa under torsion load) were lower than the yielding strength (over 150 MPa) of the cortical bone and were still within an elastic range, indicating that failure may not occur. To conclude, the grooves would increase the ability of the composite against tension and torsion loads.

**Fig. 8:**
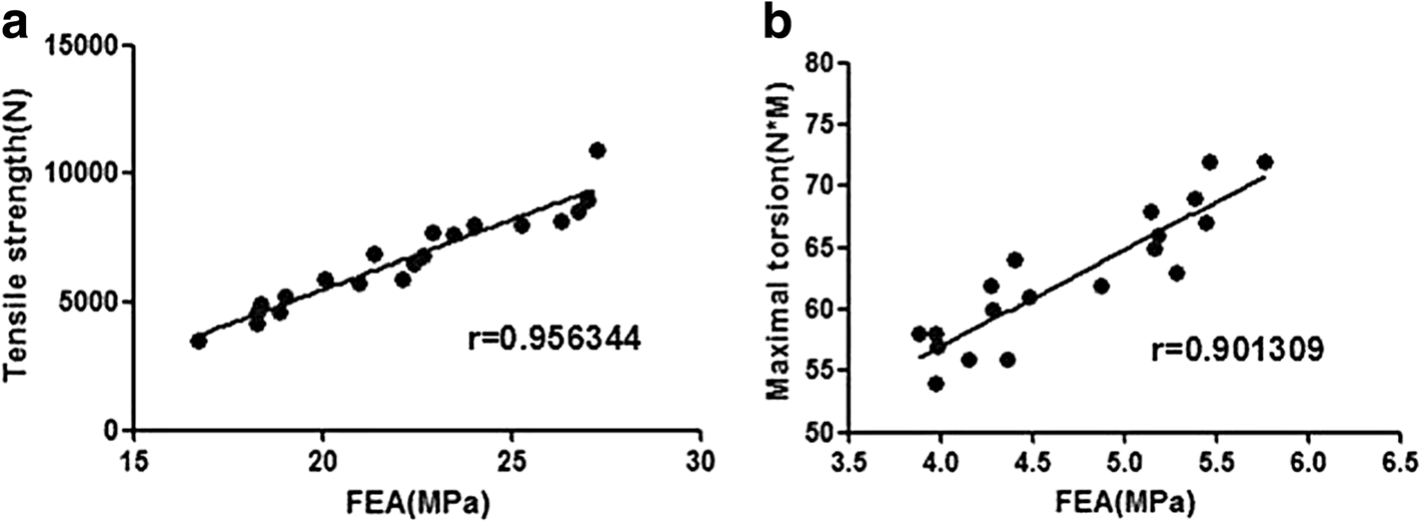
The high correlation between FEA and biomechanical experimental findings found for the strength–stiffness relation in tension (**a**) has also been noted in torsion (**b**)

The FEA results compared favorably to the biomechanical experimental tensile strength-torsion relationships of the specimens; all FEA results presented here fell within the distribution of the biomechanical experimental data (Fig. 8).

## Discussion

It is important to obtain a sufficient level of initial bone-cement interlock because some loss of interlock can be expected owing to trabecular resorption with long-term in vivo service. With 10 or more years in service, the loss of interdigitation depth was approximately 75 % [9]. Greater initial interlock between the bone and cement results in more stable constructs with less micro-motion with in vivo service [17, 18]. The quantity of interlock required for stable clinical fixation is not known, but interfaces that have micro-motion in the range of 150 μm [19] are known to result in fibrous tissue formation. The location of failure changed from the metal-cement interface for short-term implantation to the bone-cement interface for longer-term implantation. A shift in the failure mode from the implant-cement interface to the bone-cement interface with time in vivo was observed [20].

Few efforts to the morphology of the bone-cement interface in order to increase the interdigitation have been reported. Arola et al. [12] produced a variety of surface textures within the bone and demonstrated that the stability and initial displacement of the implant were dependent on the surface morphology of medullary canal. The contact area can be enlarged by reducing interfacial gaps, which can be obtained by reducing polymerization shrinkage and air and fluid inclusions [14, 15]. It might be beneficial to brush the cortical bone before cement insertion to increase the bone-cement contact area. More recent investigators have modeled bone-cement interfaces with part of a whole bone structure that included geometries of individual trabeculae determined by micro-CT [10, 21, 22]. We used self-developed reamer to create cortical grooves of the femoral inner wall and form cement circular rings in the canal wall. This increased the interlock contact area between the bone and cement. Theoretically, during infusion of bone cement, more cement will enter the inner wall of the medullary canal. The strength of the bone-cement interface can be improved through the increase of contact area and interdigitation. We investigated the difference in biomechanical behavior of the bone-cement interface in response to tension and torsion loading as a result of changing the morphology of inner wall of the bone medullary canal with grooves. The contact area of the bone-cement interface was greater for the group with cortical grooves when compared to the group without cortical grooves. The tensile strength and anti-torsion of the bone-cement interface were linearly dependent on the average contact area between the bone and cement for both tension and torsion loading conditions. There were very strong correlations between the contact area and model failure for both tensile force and maximal torsion. It suggests that achieving greater contact area of the bone-cement interface is beneficial to optimizing the interfacial strength.

Many techniques aim to improve the cement penetration between the bone and cement and to establish a durable interface. The technologies include pulsed lavage canal, place distal plug, retrograde perfusion of cement, biological type of cement, and oscillation of bone cement [23–25]. Use of pulsatile lavage and pressurization of cement has been shown to reduce the initial occurrence of radiolucent lines at the bone-cement interface [26] and increase the amount of cement penetration into the trabecular bone bed [27]. An ideal cement penetration of 3–4 mm has been proposed based on the concept that a minimum of 2 mm is needed to interlock with transverse trabeculae [28]. The penetration depth of bone cement depends on several factors, including the viscosity of cement [29], bone preparation technique [8], pressurized infusion of cement [30], and bone quality and shape, which increases the mechanical properties of bone-cement interface. Mann et al. [31] evaluated mechanical properties of the bone-cement interface under tension and the effects of bone density and bone-cement interdigitation. Increasing the volume of cement in the trabecular bone was recommended for maximizing the interface strength and reducing the incidence of interface failure. Larger cement volumes and contact between cement and cortex are both strongly negatively correlated with high trabecular bone stresses surrounding the cement and may provide a stronger support of cement mantle [32]. A large average interdigitation and contact area in trabecular bone can be achieved by preparing the bone with pulsatile lavage to allow for cement infiltration, but will be more difficult to achieve in the cortical bone. In the present study, the volume of cement was significantly greater for the specimens with grooves when compared to the specimens without grooves. The cement volume was strongly and significantly correlated with tensile force and maximal torsion, respectively. The amount of cement penetration was increased through improving the contact area which created a better initial fixing strength.

In addition, there was a very strong correlation between the contact area and model failure for both tensile force and maximal torsion. The displacement and destruction of the models occurred most at the bone-cement interface. However, the cement-prosthesis interface did not change significantly. The majority of cracks occur in the cement than the bone which is consistent with what was found experimentally [3, 10]. Porosity has been shown to affect the fatigue life of bone cements, and vacuum mixing is widely used to reduce porosity in the clinical setting [33]. In the present study, there was strongly negative correlation between the porosity and the tensile force and maximal torsion. Reduced porosity may increase the static mechanical strength of bone cement and improve the fatigue life of the cement.

As a validation, the FEA results were compared with biomechanical experimental tests. The FEA simulations compared satisfactorily with biomechanical experimental results. The high positive correlation between contact area and interface strength found for the models performed has also been noted in experimental studies of bone-cement specimens. This is consistent with the previous study [3, 10]. Combining the FEA findings with biomechanical experimental tests suggests that achieving a greater contact area between the bone and cement is essential for increasing the interfacial strength. It is believed that the increased contact area between bone and cement could enhance the initial fixation of artificial prosthesis. Our study supports the concept of obtaining sufficient initial interlock [34].

An obvious limitation of this study was that loosening of the interface was examined in an environment comprised of mechanical factors only. Complications associated with the development of interface debris and development of fibrous tissue were not considered. These factors would most likely elicit a synergistic response that potentially accelerates the loosening process with respect to results of the present study. Over the long term, bone resorption may occur at the interface, which considerably weakens the interface [35]. The study only considered time-zero biomechanics and did not consider any changes in vivo such as bone adaptation or resorption. Additional experimental work is needed to delineate the role of micromechanical behavior and gross structural response under fatigue loading. Another recognized limitation of the study was the limited number of specimens prepared and examined. Nevertheless, this study has examined mechanical properties of the bone-cement interface and distinguished that the bone surface morphology contributes to increasing the interlock of the bone-cement interface.

## Conclusions

The majority of cracks occurred in the cement than the bone. Changing the surface morphology of the bone medullary canal from a smooth bore cortical tube to one with grooves would make a better interlocking between the bone and cement, which may potentially increase the initial stability and biomechanical strength of the bone-cement interface, and prolongs the survival life of prosthesis.
